# Prevalence of prolonged grief disorder and its symptoms in Chinese parents who lost their only child: A systematic review and meta-analysis

**DOI:** 10.3389/fpubh.2022.1016160

**Published:** 2022-09-27

**Authors:** Meng-Di Yuan, Zong-Qin Wang, Lei Fei, Bao-Liang Zhong

**Affiliations:** ^1^Research Center for Psychological and Health Sciences, China University of Geosciences (Wuhan), Wuhan, China; ^2^Department of Psychiatry, Wuhan Mental Health Center, Wuhan, China; ^3^Department of Clinical Psychology, Wuhan Hospital for Psychotherapy, Wuhan, China; ^4^Department of Ultrasound, Renmin Hospital, Hubei University of Medicine, Shiyan, China

**Keywords:** prolonged grief disorder, *Shidu* parents, prevalence, meta-analysis, China

## Abstract

**Background:**

Parents who lost their only child and cannot have a second child (“*Shidu*”) have been a large population in China. Prolonged grief disorder (PGD) in *Shidu* parents is of clinical and public health concern but the reported PGD prevalence varies widely. To facilitate the planning of grief counseling services, this meta-analysis estimated prevalence of PGD and its symptoms and identified subgroups at elevated risk for PGD.

**Methods:**

We searched English and Chinese literature databases to identify cross-sectional surveys reporting prevalence of PGD or PGD symptoms in Chinese *Shidu* parents. The Joanna Briggs Institute Critical Appraisal Checklist for Studies Reporting Prevalence Data (“JBI”) was used to assess risk of bias of included studies.

**Results:**

Seven studies with a total of 2,794 *Shidu* parents were included and their JBI scores ranged from five to eight. The pooled prevalence of PGD and PGD symptoms was 20.9% and 75.0%, respectively. Greater risk of PGD was observed in mothers [vs. fathers, OR (odds ratio) = 1.89, *P* = 0.001] and in parents with religious beliefs (vs. without religious beliefs, OR = 1.65, *P* = 0.040). More severe PGD symptoms were presented in parents whose only child died from accidents [vs. illness, MD (mean difference) = 3.99, *P* < 0.001]. Deceased children of PGD parents were older than those of non-PGD parents (MD = 1.64, *P* = 0.035) and PGD parents had a shorter duration since the loss than non-PGD parents (MD = −3.26, *P* = 0.013).

**Conclusions:**

PGD is prevalent among *Shidu* parents. Grief counseling services for *Shidu* parents would be more effective if they target those who are mothers and have religious beliefs and those whose children died from accidents, lost children are older, and loss occurs more recently.

## Introduction

In China, the one-child policy in the past four decades has created tens of millions of one-child families (1). The estimated number of one-child families in China still reached 250 million in 2020 despite the abolition of the one-child policy in 2015 (2). The one-child policy, while it effectively slows the population growth, still produces a large number of couples who lost their only child due to various reasons. In China, “*Shidu* parents” are an official term used to describe couples who lost their only child and cannot have a second child because the mothers have passed the reproductive age (i.e., 50 years and above) or the couples are not willing to adopt children (3). According to Chinese demographers, the number of *Shidu* parents had been 1 million in 2018 and, due to population growth and aging, there will be up to 8 million *Shidu* parents by 2030 in China (4–6).

Grief is a normal emotional reaction following the death of a close friend or family member, however, some bereaved individuals may be overwhelmed and develop prolonged grief disorder (PGD), which is characterized by intense and persistent feelings of grief that are substantially prolonged beyond culturally normed expectations and causes clinically significant distress or impairment in social, occupational, or other important domains of functioning (7). In empirical studies with mixed samples of bereaved persons such as those who lost their parents, spouses, children, and other relatives, prevalence rates of PGD are 1.8–13.9% in China with the most severe PGD symptoms being observed in Chinese persons who lost their children and 9.8% in a meta-analysis of worldwide prevalence studies with higher prevalence of PGD being observed in persons who lost their siblings in western countries (8–10).

In the context of Chinese culture, death of the only child is particularly devastating for the *Shidu* parents because the tradition of loyalty and filial piety emphasizes that “no offspring is the worst one of the three acts that a person is not filial to his/her ancestors” and the parents are likely to feel discrimination and perceive stigma for failure to continue the family line (11, 12). On the other hand, because of the tradition of “bringing up children to support parents in their old age”, the loss of the only child means the loss of the primary home care provider for elderly parents or parents when they get old, in particular for the rural parents (13, 14). As a result, traumatic experiences of Chinese *Shidu* parents may be complicated by these negative psycho-socio-cultural factors, which may further magnify the risk of PGD in this vulnerable subpopulation. Accurately gauging the prevalence of PGD in *Shidu* parents and identifying subgroups at high risk for PGD are essential for the mental health planning and policy-making for *Shidu* parents.

In China, a few empirical studies have investigated the prevalence of PGD in *Shidu* parents, but these studies reported a wide range of prevalence estimates (i.e., 9.5–35.3% for PGD and 28.9–95.7% for PGD symptoms) (15–18) and inconsistent findings on the high-risk subgroups of PGD according to parents' socio-demographic and bereavement-related characteristics. For example, Wang found comparable prevalence of PGD between *Shidu* mothers and fathers (10.3% vs. 8.5%, *P* = 0.500) but Zhang and colleagues found significantly higher prevalence of PGD in *Shidu* mothers than fathers (27.8% vs. 13.6%, *P* = 0.041) (15, 19); Zhang found significantly less severe PGD symptoms in *Shidu* parents who lost boys than those who lost girls (*P* = 0.006) but Wang found similar PGD symptom severity between *Shidu* parents who lost boys and those who lost girls (*P* = 0.846) (15, 20). One of the potentially important reasons for the above controversy is the small sample sizes of available studies, which may result in unstable estimates of prevalence and findings on high-risk subgroups of PGD. To facilitate the development of preventive strategies for PGD and the delivery of grief counseling services, it is necessary to perform a systematical review and meta-analysis to provide accurate estimates of the prevalence of PGD and PGD symptoms in Chinese *Shidu* parents.

## Methods

This systematic review and meta-analysis was reported in accordance with the Preferred Reporting Items for Systematic Reviews and Meta-Analyses guidelines (PRISMA) (Supplementary materials: Appendix 1). The first and second authors in dependently performed the literature search, assessed the eligibility of retrieved studies, extracted data from included studies, and evaluated the risk of bias (RoB) of included studies. Any disagreements were resolved by the corresponding author.

### Literature retrieval

We conducted a literature search in both Chinese and English bibliographic databases from their inception to 7 September, 2022: China National Knowledge Infrastructure, Wanfang data, SinoMed, VIP Information, PubMed, Embase, and PsycInfo. The search terms were as follows: (Shidu^*^ OR “only child” OR bereave^*^ OR “family planning”) AND (grief OR mourning OR sorrow) (Supplementary materials: Appendix 2). Reference lists of the included studies were also searched manually to identify additional studies.

### Inclusion and exclusion criteria

Cross-sectional studies that reported the prevalence of PGD or PGD symptoms in Chinese parents who suffered the loss of their only child and had no a second child were eligible for this study. The presence of PGD or PGD symptoms was assessed by using validated instruments such as the 13-item Prolonged Grief Disorder (PG-13) and Inventory of Complicated Grief. Studies that did not directly provide a prevalence estimate of PGD or PGD symptoms but provided other data that could be used to calculate prevalence estimates were also included. If two or more publications from the same sample were available, only those with the most complete data were included. Studies focusing on loss other than the death of the only child were excluded.

### Data extraction

Variables extracted from included studies were first author, publication year, study site, survey date, sampling method, sample size, mean age of the study sample, percentage of females in the study sample, prevalence of PGD or PGD symptoms, and assessment of PGD or PGD symptoms. Because some of our included studies presented and compared PGD prevalence rates and scores of PGD symptoms [as measured by the 11 PGD symptom items of the PG-13 (“PG-11 score”)] between subgroups according to some socio-demographic and bereavement-related factors, we also extracted these comparative data to ascertain subgroups at elevated risk for PGD and having more severe PGD symptoms [i.e., means and standard deviations (SDs) of PG-11 scores and numbers of subjects by fathers and mothers, respectively]. These factors included age, sex, marital status, and religious beliefs of parents and age, sex, causes of death, and duration since the death of the decreased.

### RoB assessment of included studies

We used the Joanna Briggs Institute Critical Appraisal Checklist for Studies Reporting Prevalence Data (“JBI checklist” hereafter) to assess the level of RoB of included studies (21). The JBI checklist has nine items that are designed to assess three methodology domains of an individual study: sample representativeness (sample frame, sampling method, sample size, description of participants and setting), statistics (sample coverage of the data analysis, statistical analysis, response rate), and accuracy of the outcome assessment (validity of the instrument for assessing the outcome, standardization and reliability of the instrument for assessing outcome) (22, 23). Each item has four answer options (yes, no, unclear, not applicable) and one point is allocated for a “yes” response, which yields a “0–9” total RoB score for a study with a higher score denoting lower RoB.

### Statistical analysis

Prevalence data were synthesized by using the “metaprop” module of R, version 4.2.0, which adopted the one-step generalized linear mixed models with the logit link function (23). We used I2 statistic to test statistical heterogeneity between studies. When I2 >50%, a random-effects model was used to calculate the combined estimate of prevalence; otherwise, a fixed-effects model was adopted. We used subgroup analysis to explore the source of heterogeneity in the prevalence estimates. Publication bias was assessed by funnel plots and Begg's test. Two-sided *P* < 0.05 was set as statistically significant.

With regard to the meta-analysis of comparative data, we used the “metabin” and “metacont” modules of R, version 4.2.0, for dichotomous and continuous outcomes, and the corresponding effect size measures were odds ratios (ORs) and mean differences (MDs), respectively.

## Results

**Figure 1:**
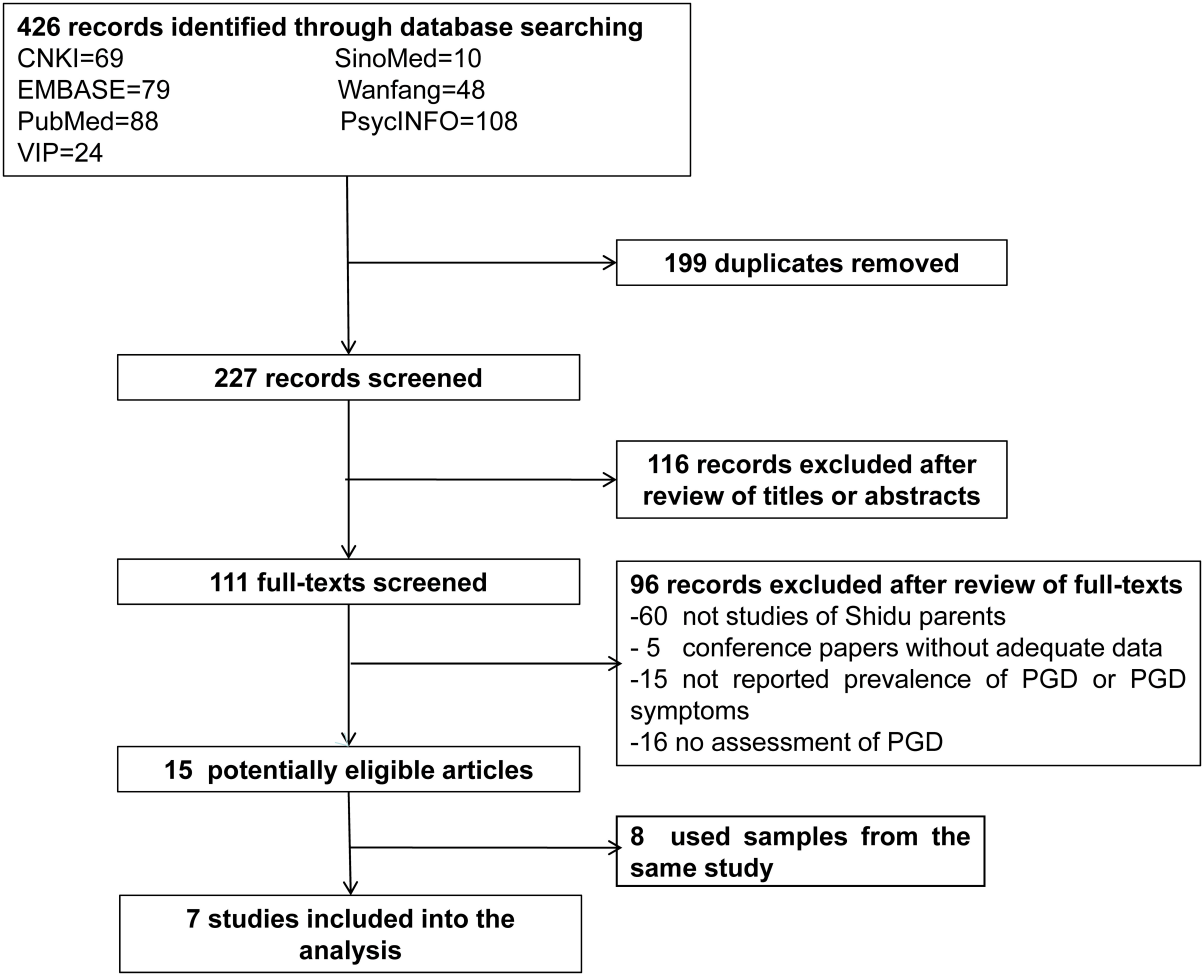
Flowchart of study inclusion.

Finally, seven studies with a total of 2,794 *Shidu* parents were included: one focused on PGD symptoms only (18), five focused on PGD only (15, 16, 24–26), and one focused on both PGD and PGD symptoms (19). The flowchart of study inclusion and detailed characteristics of included studies are shown in Figure 1 and Table 1, respectively. Four studies assessed the presence of PGD by using the PG-13 based on the Prigerson et al. (27) diagnostic criteria while two assessed the presence of PGD by using a self-developed scale based on the International Classification of Diseases 11th Revision (ICD-11) (15, 16, 19, 24–26).

**Table 1:** Characteristics of included studies.

**Study**	**Reference**	**Survey site**	**Survey period**	**Number of survey completers**	**Sampling method**	**Response rate (%)**	**Numbers of men and women**	**Mean age of the sample (years)**	**Instrument**	**Survey method**	**Diagnostic criteria of prolonged grief disorder (PGD)**	**Number of subjects with PGD (%)**	JBI checklist score*
Xu et al. (2014)	(18)	Dujiangyan	October 2010—March 2011	116	Cluster sampling	53.7	0/116	39.4	Inventory of Complicated Grief (ICG)	Self-report	ICG>25	PGD symptoms: 111 (95.7)	6
Zhang & Jia et al. (2019)	(24)	Beijing, Zhengzhou, Haerbin, Chongqing, Baotou	June -December 2017	466	Convenience sampling	93.8	212/254	60.2	Prolonged Grief Disorder-13 (PG-13)	Self-report	Prigerson et al. (27)	109 (23.4)	7
Wang et al. (2020)	(15)	5 urban districts in Shenyang	March -September 2017	483	Two-stage cluster sampling	81.2	201/282	61.98	PG-13	Self-report	Prigerson et al. (27)	46 (9.5)	7
Zhang et al. 2020	(19)	1 district in Shanghai	September 2015 - January 2017	149	Stratified random sampling	96.1	59/90	62.3	PG-13	In-person interview	Prigerson et al. (27) PG-11>36	33 (22.2) PGD symptoms: 43 (28.9)	8
Zhou et al. 2020	(16)	24 cities from 8 provinces and 3 municipalities	April 2017 - May 2018	1,030	Convenience sampling	97.7	381/643	59.9	ICD-11 diagnostic algorithm, constructed based on existing scales	Online and in-person interview	ICD-11	366 (35.5)	8
Ma et al. 2022	(26)	20 communities in Sujiatun district in Shenyang	November 2019—February 2020.	240	Two-stage cluster sampling	87.3	113/127	62.9	PG-13	Self-report	Prigerson et al. (27)	28 (11.7)	8
Xu et al. 2022	(25)	Yanji and Haerbin	December 2019—January 2020	310	Convenience sampling	Not reported	92/218	61.71 ± 5.48	ICD-11 diagnostic algorithm, constructed based on existing scales	Self-report	ICD-11	102 (32.9)	5

^*^The joanna briggs institute critical appraisal checklist for studies reporting prevalence data.

The JBI checklist scores of the seven included studies ranged from five to eight (Table 1). No study was rated nine. The two most common methodological problems of included studies were unstandardized way of outcome measurement (*n* = 4) and inappropriate sampling method (*n* = 3) (Supplementary materials: Appendix 3).

**Figure 2:**
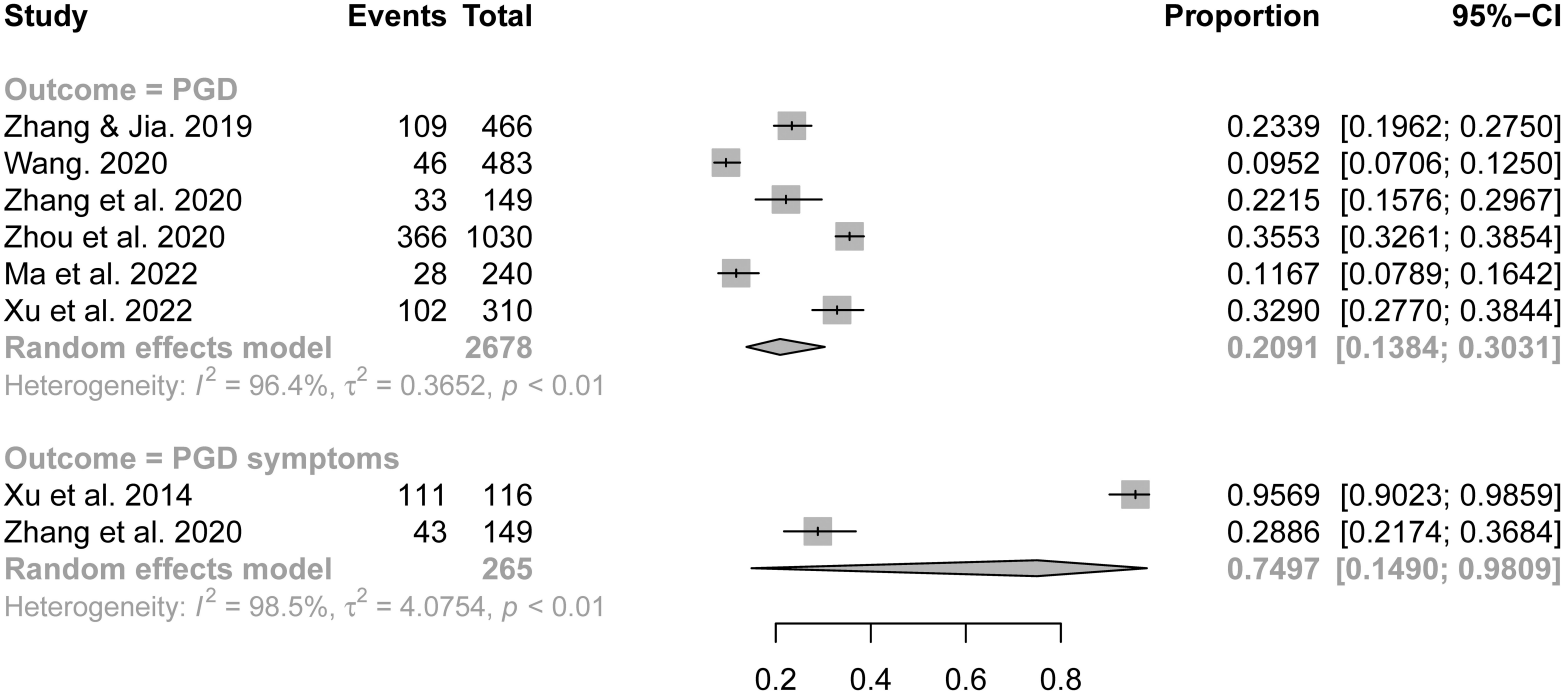
Forest plot of prevalence of prolonged grief disorder and its symptoms.

The synthesized prevalence of PGD and its symptoms were 20.9% (95% CI: 13.8–30.3%) and 75.0% (95% CI: 14.9–98.1%), respectively (Figure 2). Subgroup analyses identified four factors as statistically significant sources of heterogeneity in the prevalence rates of PGD across included studies (Table 2): % of mothers in the survey sample, sampling method, diagnostic criteria of PGD, and JBI checklist score. Specifically, significantly higher prevalence of PGD was observed in studies with a percentage of mothers >60% than those with a percentage of mothers ≤ 60% (30.9% vs. 14.0%, *P* = 0.001), in studies adopted convenience sampling than those adopted probability sampling (30.5% vs. 13.4%, *P* < 0.001), in studies diagnosed PGD with ICD-11 than those diagnosed PGD with Prigerson et al. 2009 criteria (34.9% vs. 15.7%, *P* < 0.001), and in studies with JBI scores of “7-8” than those with a JBI score of “5” (32.9% vs. 18.9%, *P* = 0.013).

**Table 2:** Subgroup analyses of meta-analysis of prevalence of Prolonged Grief Disorder (PGD) according to study-level factors.

**Study-level factor**	**Number of studies**	**Number of *Shidu* parents**	**Number of parents with PGD**	**Heterogeneity, I2 (%)**	**Pooled prevalence (95% CI), %**	**Q**	***P***
% of mothers in the study sample*
≤ 60%	3	1,189	183	94.5	14.0 (8.7, 21.9)		
>60%	3	1,489	501	80.5	30.9 (24.9, 37.6)	10.29	0.001
Mean age (years) of study sample
≤ 61	2	1,496	475	95.4	29.3 (21.6, 38.5)		
>61	4	1,182	209	95.9	17.3 (10.2, 27.9)	3.29	0.070
Sampling method
Convenience	3	1,806	577	90.7	30.5 (24.7, 37.0)		
Probability	3	872	107	87.6	13.4 (8.7, 20.0)	13.2	<0.001
Way of instrument administration
Self-administration	4	1,259	257	96.0	17.7 (10.4, 28.4)		
Interview	2	1,179	399	90.1	29.4 (20.8, 39.7)	2.82	0.093
Diagnostic criteria of PGD
Prigerson et al. (27)	4	1,338	216	92.4	15.7(10.5, 22.8)		
ICD-11	2	1,340	468	0.0	34.9 (32.4, 37.5)	71.51	<0.001
JBI checklist score**
7–8	5	2,368	582	97.0	18.9 (12.0, 28.5)		
5	1	310	102	Not applicable	32.9 (27.7, 38.4)	6.140	0.013

*The two subgroups were generated by median split of the % of mothers in the study sample of included studies.

**The joanna briggs institute critical appraisal checklist for studies reporting prevalence data.

Begg's test (z = −1.32, *P* = 0.189) revealed no statistically significant publication bias across studies included for the meta-analysis of prevalence of PGD (Supplementary materials: Appendix 4).

Results of meta-analyses comparing the PGD prevalence and PGD symptom scores between different subgroups (Supplementary materials: Appendices 5, 6) show that, in comparison to *Shidu* fathers, *Shidu* mothers had higher risk of PGD (OR = 1.89, *P* = 0.001) and more severe PGD symptoms (MD = 2.60, *P* = 0.039). *Shidu* parents who endorsed religious beliefs were more likely to have PGD than those who did not have religious beliefs (OR = 1.65, *P* = 0.040). Risk of PGD (OR = 1.01, *P* = 0.937) and severity of PGD symptoms (MD = −0.85, *P* = 0.524) did not differ significantly between parents who lost boys and girls. Parents whose only child died from accidents had statistically more severe PGD symptoms than those whose only child died from illness (MD = 3.99, *P* < 0.001).

Results of meta-analyses comparing age of the deceased and years since the death of the deceased (Supplementary materials: Appendix 7) show that the deceased children's age was significantly older in PGD than non-PGD parents (MD = 1.64, *P* = 0.035) and the duration since the loss of the PGD parents was significantly shorter than non-PGD parents (MD = −3.26, *P* = 0.013).

## Discussion

This study used available meta-analyzable data from the literature to estimate the prevalence of PGD and its symptoms and identify subgroups that are at elevated risk of PGD and have more severe PGD symptoms among Chinese *Shidu* parents. We found that as high as 75.0% of the *Shidu* parents had PGD symptoms and 20.9% of the *Shidu* parents met the diagnostic criteria for PGD. Greater risk of PGD was observed in *Shidu* mothers (vs. fathers) and in parents with religious beliefs (vs. without religious beliefs). More severe PGD symptoms were observed in *Shidu* mothers (vs. fathers) and parents whose only child died from accidents (vs. illness). In addition, PGD parents' deceased children were more likely to be older than those of non-PGD parents and PGD parents had a shorter duration since the loss of the only child than non-PGD parents.

The 75.0% prevalence of PGD symptoms in Chinese *Shidu* parents is much higher than the 13.9–35.0% prevalence of PGD symptoms in the general Chinese population who experienced the loss of family members (28). The 20.9% prevalence of PGD in *Shidu* parents is also higher than the 1.8–13.9% prevalence of PGD in Chinese bereaved adults and the 9.8% worldwide prevalence of GPD in bereaved adults (8–10). In western countries, couples who lose their only child and have no remaining children are at significantly higher risk of grief than those with one or more surviving children and the grief in these parents is more likely to be chronic and long-lasting (29, 30). In the Chinese society, the loss of the only-child does not only mean “no descendant” and “will die without the care of natural sons/daughters at the bedside” for *Shidu* parents, but also is associated with a variety of challenges including the economic difficulties, the collapse of the family structure, and the loss of joy and hope (31). The high prevalence of PGD symptoms and PGD in our meta-analysis confirms the elevated risk of PGD in Chinese *Shidu* parents.

The higher prevalence of PGD and greater severities of PGD symptoms in bereaved mothers than fathers are quite consistent in the literature (32–34). Our meta-analysis replicated the gender differences in grieving in the population of Chinese *Shidu* parents. There are several explanations for the gender differences in the experiences, expression, and symptom development of grief. For example, bereaved mothers are more likely to share their feelings of grief with others, which helps them process and work through their grief; however, due to men's social roles as “providers and protectors”, bereaved fathers tend to move past the loss to make their lives move forward instead of talking about the loss (33–36). As a supporting case in point, findings from a longitudinal study has suggested that prolonged grief in men is more likely to be an acute and decreasing reaction, whereas in women it is more likely to be a delayed and accumulating grief action (37). In the context of China's tradition of “Men's work centers around outside while women's work centers around the home”, mothers are more involved in the children's care than fathers (38, 39). We speculate that the higher prevalence of PGD and more severe PGD symptoms in *Shidu* mothers may be due to their much more efforts made for and much more time spent on the care of the only child, which goes for nothing after the loss. This “mother predominance” phenomenon of PGD is further supported by higher PGD prevalence in studies with female-predominant samples (>60% vs. ≤ 60%) in the subgroup analysis (Table 2).

In western countries, religious beliefs are often associated with lower risk of mental health problems including grief (40, 41). Nevertheless, in China, the world's most atheistic country, believing in religion is a rare phenomenon and many Chinese people become religious believers for the purpose of seeking help from the religion after they suffer from emotional problems and other difficulties (42, 43). The elevated risk of PGD in *Shidu* parents with religious beliefs in our study might be ascribed to their help-seeking behaviors from the religion, that is, religious beliefs might be the result of PGD. Because the included cross-sectional studies cannot provide evidence on the chronological order of religious beliefs and PGD, longitudinal data are needed to further clarify the causal PGD-religion relationship. In this study, death from accidents (vs. illness) was associated with more PGD symptoms in *Shidu* parents. This finding is similar to the significant association between the unexpected death and higher level of grief in bereaved individuals in western countries, since the sudden and unexpected loss damages parents' sense of parenthood and other layers of social identity and destroys the parents' feeling of safety and security (29, 44, 45).

“White-haired parents' attendance of the funeral of the black-haired young children” is the most painful tragedy in traditional Chinese culture (46). Because older adult children are usually have stable jobs and family lives but younger adult children are often in early stage of the career before their deaths, the death of older adult children takes away more things than younger adult children, including parents' efforts in bringing up the children. This might explain the older age of the decreased children in *Shidu* parents with PGD. Similarly, in the Netherlands, Wijngaards-de Meij and colleagues reported the positive association between deceased child' age and severe grief reactions in bereaved couples following the death of their children [29), which might be attributed to older deceased children's parents' more times and efforts put into rearing children and lower potential for producing future offspring. In prior studies of grief in bereaved persons in both Asian and western countries, there is a negative association between time since loss and the level of grief (29, 34, 36, 47). Our finding on the shorter duration since the loss in PGD than non-PGD *Shidu* parents is consistent with the universal phenomenon around the world, that is, grief symptoms become milder over time.

The finding on the comparable prevalence of PGD and similar levels of PGD symptoms between *Shidu* parents of boys and girls is not expected, because the traditional Chinese culture prefers sons to daughters (48). Although the loss of the only boy means the discontinuation of the family line, the intimate relationship is generally closer for parents-daughters than parents-boys in China (49), possibly resulting in the similar PGD risk and similar levels of PGD symptoms between parents who lost boys and girls.

This study has several limitations. First, the number of eligible studies for this meta-analysis is small, in particular studies of PGD symptoms. Second, none of the included studies were completely free from risk of bias. Because subgroup analyses reveal that studies with convenience sampling and low JBI checklist scores tended to report higher prevalence of PGD (Table 2), we may have overestimated the true prevalence of PGD in Chinese *Shidu* parents. Third, the higher prevalence of PGD in studies with ICD-11 than those with Prigerson et al. 2009 criteria in the subgroup analysis (Table 2) suggests that the true prevalence of PGD may be underestimated if the ICD-11 was adopted by all included studies. Fourth, these included studies provided few data on the mental health services utilization of *Shidu* parents, which are important for the planning for the grief counseling services for this vulnerable population. More studies that recruit representative samples of Chinese *Shidu* parents and diagnose PGD according to international standardized diagnostic criteria such as ICD-11 and DSM-V are warranted.

In China, the grief counseling services are still in their early stage of development. The limited grief counseling service resources are mainly concentrated in the limited number of palliative care hospitals and departments of palliative care in general hospitals. The other challenges to the grief counseling service system include inadequate professionals engaged in grief counseling, lack of standardized training and supervision of these professionals, lack of community-based screening and referral system for PGD individuals, and concerns on the quality of counseling services provided (50). Given the large number of *Shidu* parents in China and the 75.0% PGD symptoms prevalence and the 20.9% PGD prevalence in this population, the level of potentially unmet need for grief counseling services is very high. Our study highlights the urgent needs for strengthening the grief counseling services for this population. Possible public health strategies may consider the establishment of the primary care-based two-way referral system for *Shidu* parents with PGD, the training and supervision of community health and social workers to help them acquire the basic capacity to screen for and manage PGD, the provision of outreach services by mental health workers and clinical psychologists from hospitals, the integration of counseling services into the community services, and professional-sponsored organization of *Shidu* parents to provide the avenue for group therapy.

The grief counseling services for *Shidu* parents may include psychosocial support to improve psychological well–being, periodical screening for PGD and other mental health problems, and psychiatric referral and treatment when necessary. Further, these services for *Shidu* parents would be more effective if they target those who are mothers and have religious beliefs and those whose only child died from accidents, lost children are older, and loss occurs more recently.

## Data availability statement

The original contributions presented in the study are included in the article/Supplementary material, further inquiries can be directed to the corresponding author.

## Author contributions

B-LZ: design of the study, interpretation of data for the study, revising the paper critically for important intellectual content, and final approval of the version to be submitted. M-DY: acquisition and analysis of data for the study, drafting the paper, revising the paper for important intellectual content, and interpretation of data for the study. Z-QW: acquisition and analysis of data for the study, drafting the paper, and interpretation of data for the study. LF: acquisition and analysis of data for the study, drafting the paper, and revising the paper for important intellectual content. All authors contributed to the article and approved the submitted version.

## Funding

This work was supported by National Natural Science Foundation of China (Grant Number: 71774060), 2015 Irma and Paul Milstein Program for Senior Health Awards from the Milstein Medical Asian American Partnership Foundation, and Wuhan Health and Family Planning Commission (Grant Numbers: WX17Q30, WG16A02, and WG14C24). The funding source listed had no role in study design; in the collection, analysis and interpretation of data; in the writing of the report; and in the decision to submit the paper for publication.

## Conflict of interest

The authors declare that the research was conducted in the absence of any commercial or financial relationships that could be construed as a potential conflict of interest.

## Publisher's note

All claims expressed in this article are solely those of the authors and do not necessarily represent those of their affiliated organizations, or those of the publisher, the editors and the reviewers. Any product that may be evaluated in this article, or claim that may be made by its manufacturer, is not guaranteed or endorsed by the publisher.
